# Massive hemothorax due to bleeding from thoracic spinal fractures: a case series and systematic review

**DOI:** 10.1186/s13049-020-00783-0

**Published:** 2020-09-11

**Authors:** Kohei Ninomiya, Akira Kuriyama, Hayaki Uchino

**Affiliations:** Emergency and Critical Care Center, Kurashiki Central Hospital, 1-1-1 Miwa, Kurashiki, Okayama 710-8602 Japan

**Keywords:** Wounds and injuries, Hemothorax, Spinal fractures

## Abstract

**Background:**

Massive hemothorax secondary to thoracic spinal fractures is rare, and its clinical characteristics, treatment, and prognosis are unknown. We present two cases of thoracic spinal fracture-induced massive hemothorax and a systematic review of previously reported cases.

**Methods:**

This study included patients with traumatic hemothorax from thoracic spinal fractures at a Japanese tertiary care hospital. A systematic review of published cases was undertaken through searches in PubMed, EMBASE, and ICHUSHI from inception to October 13, 2019.

**Results:**

Case 1: An 81-year-old man developed hemodynamic instability from a right hemothorax with multiple rib fractures following a pedestrian–vehicle accident; > 1500 mL blood was evacuated through the intercostal drain. Thoracotomy showed hemorrhage from a T8-burst fracture, and gauze packing was used for hemostasis. Case 2: A 64-year-old man with right hemothorax and hypotension after a fall from height had hemorrhage from a T7-burst fracture, detected on thoracotomy, which was sealed with bone wax. Hypotension recurred during transfer; re-thoracotomy showed bleeding from a T7 fracture, which was packed with bone wax and gauze for hemostasis. The systematic review identified 10 similar cases and analyzed 12 cases, including the abovementioned cases. Inferior part of thoracic spines was prone to injury and induced right-sided hemothorax. Most patients developed hemodynamic instability, and some sustained intra-transfer hemorrhage; direct compression (gauze packing, bone wax, and hemostatic agents) was the commonest hemostatic procedure. The mortality rate was 33.3%.

**Conclusions:**

Hemothorax due to thoracic spinal fracture can be fatal. Thoracotomy with direct compression is necessary in hemodynamically unstable patients.

## Background

Chest injuries occur in approximately 60% of polytrauma patients and account for 20–25% of trauma-related mortality [1]. Two large observational studies from Level 1 trauma centers in the United States and China suggested hemothorax as a complication in 3.6 to 5.9% of all chest trauma, with associated mortality rates of 1.7–9.4% [2, 3]. Most cases of hemothorax can be usually managed by observation or tube thoracostomy alone. However, the available evidence suggests that chest trauma might be associated with poor prognosis.

Hemothorax occurs from an injury to the pulmonary parenchyma, hilar vessels, heart (with a communicating defect between the pericardium and pleura), great vessels (opening into the pleura), intercostal vessels, or internal thoracic arteries [4]. Moreover, the thoracic spine constitutes a part of the chest. Spinal fracture is a common chest trauma- associated injury that has a reported prevalence of 19% [2]. However, hemothorax due to spinal fracture has rarely been reported.

We report two cases of life-threatening, massive hemothorax due to bleeding from thoracic spinal fractures that was diagnosed during an emergency-room thoracotomy (ERT) undertaken as damage-control surgery (DCS). Given the rarity of this entity, we summarize the evidence on clinical characteristics, treatment, and prognosis of hemothorax with a systematic review of similar cases.

## Methods

The Kurashiki Central Hospital is a 1131-bed tertiary care hospital located in Kurashiki city, Okayama Prefecture, in western Japan. The institution covers the medical district of the southwestern area of Okayama Prefecture, with approximately 800,000 residents. Approximately 60,000 patients visit the emergency department every year. The institution annually treats 400 trauma patients, with 160 severe trauma cases (Injury Severity Score [ISS] > 15) on average. We reviewed the institutional database and electronic medical charts of trauma patients to identify patients with massive hemothorax due to bleeding from thoracic spinal fractures, from July 1, 2013, to June 30, 2019. Patient characteristics and clinical outcomes were recorded. Informed consent for publication was obtained from the patients or their legal guardians.

There is no standardized systematic approach for the diagnosis and treatment of these cases. Therefore, we conducted a systematic review of previously published case reports and series on hemothorax. We adhered to the Preferred Reporting Items for Systematic Reviews and Meta-Analyses statement for reporting systematic reviews [5]. Our review protocol was registered in the University hospital Medical Information Network Center registry (UMIN 000038254). We included case reports or case series on hemothorax where a breach of the thoracic vertebrae was confirmed as the bleeding site. We excluded case repots or case series that were published in non-peer-reviewed journals, those without details after author contact attempts, and those on hemothorax where a breach of the thoracic vertebrae was not the bleeding site. We searched Medline, EMBASE, and ICHUSHI (“Igaku CHUo zasSHI,” meaning “Medical Central Journals” in Japanese) databases without language restrictions to enhance the generalizability of our findings [6, 7] (Supplementary Table 1). Furthermore, we checked the reference lists of the included articles for potentially relevant reports. Our search was updated on October 13, 2019.

Two authors (KN and AK) independently assessed and duly verified the eligibility of published cases for inclusion in the review. Disagreements, if any, were resolved through discussion with the third author (HU). We contacted the original authors for details of missing information as relevant for the assessment of eligibility to our review or data for analysis. We used descriptive statistics to summarize clinical characteristics, treatment, and prognosis of hemothorax following bleeding from thoracic spinal fractures.

## Results

A total of 708 patients with thoracic injury and 231 patients with hemothorax were treated at the study center from July 1, 2013, through June 30, 2019. Two patients, comprising 0.3% of all thoracic injuries and 0.9% of all hemothorax, had hemothorax from bleeding caused by thoracic spinal injuries.

### Case 1

**Fig. 1:**
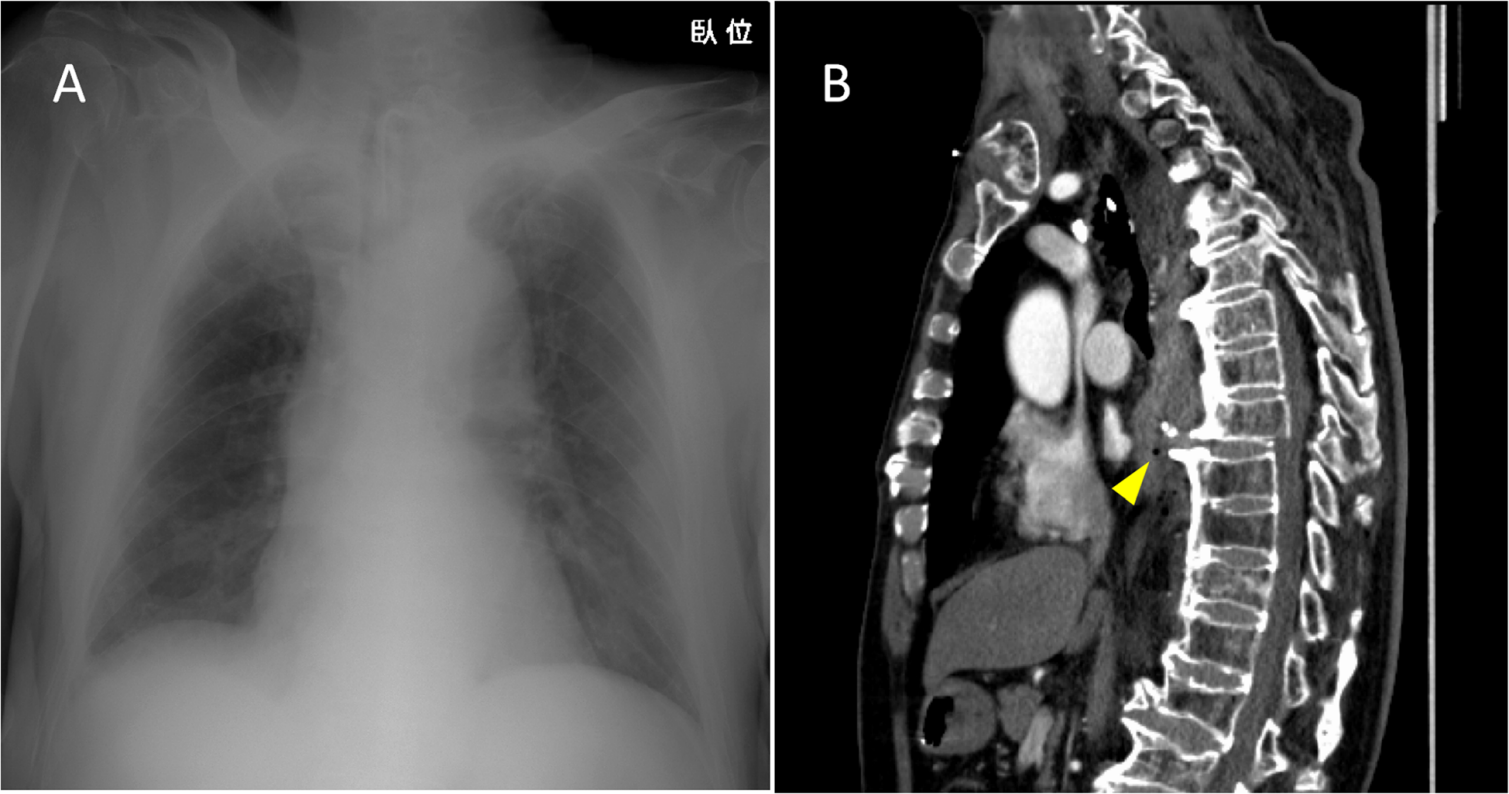
Images from Case 1. **a** Chest radiography shows extended opacity of the right chest, and **b** computed tomography shows a T8-burst fracture (arrow head)

An 81-year-old man sustained injuries in a pedestrian–vehicle accident and was hospitalized in a hemodynamically unstable state with decreased chest sounds on the right side. Focused sonographic assessment for trauma was negative. Chest radiography revealed chest opacity and multiple rib fractures on the right side, which indicated a right massive hemothorax (Fig. 1a). A pelvic fracture was suspected from the pelvic radiograph. Continuous hemorrhage (> 1500 mL) evacuated through an intercostal drain and hemodynamic instability necessitated an ERT (right anterolateral thoracotomy). An active bleeding site was not initially identified; therefore, pre-peritoneal pelvic packing was undertaken, given the possibility of retroperitoneal hemorrhage from the pelvic fracture; however, the retroperitoneal hematoma was small, and pelvic packing did not improve the patient’s hemodynamic status. The right thorax was re-explored and active bleeding from a T8-burst fracture was identified; no other source of bleeding was detected. Temporary gauze packing was effective, and the patient was transferred to the intensive care unit (ICU). Computed tomography (CT) scan revealed traumatic brain injury (TBI), C5 fracture, bilateral clavicular fractures, right multiple rib fractures, T8-burst fracture with complete neurological deficit, pelvic fracture, and limb fractures (Fig. 1b), with an ISS of 57. Spinal fixation was not undertaken because of impaired consciousness and complete paraplegia, in accordance with the preference of the patient’s family members. The patient developed complicated pneumonia, which was treated with antibiotics and remained in the ICU for 30 days, with an otherwise uneventful course and was subsequently transferred to a rehabilitation hospital on Day 48.

### Case 2

**Fig. 2:**
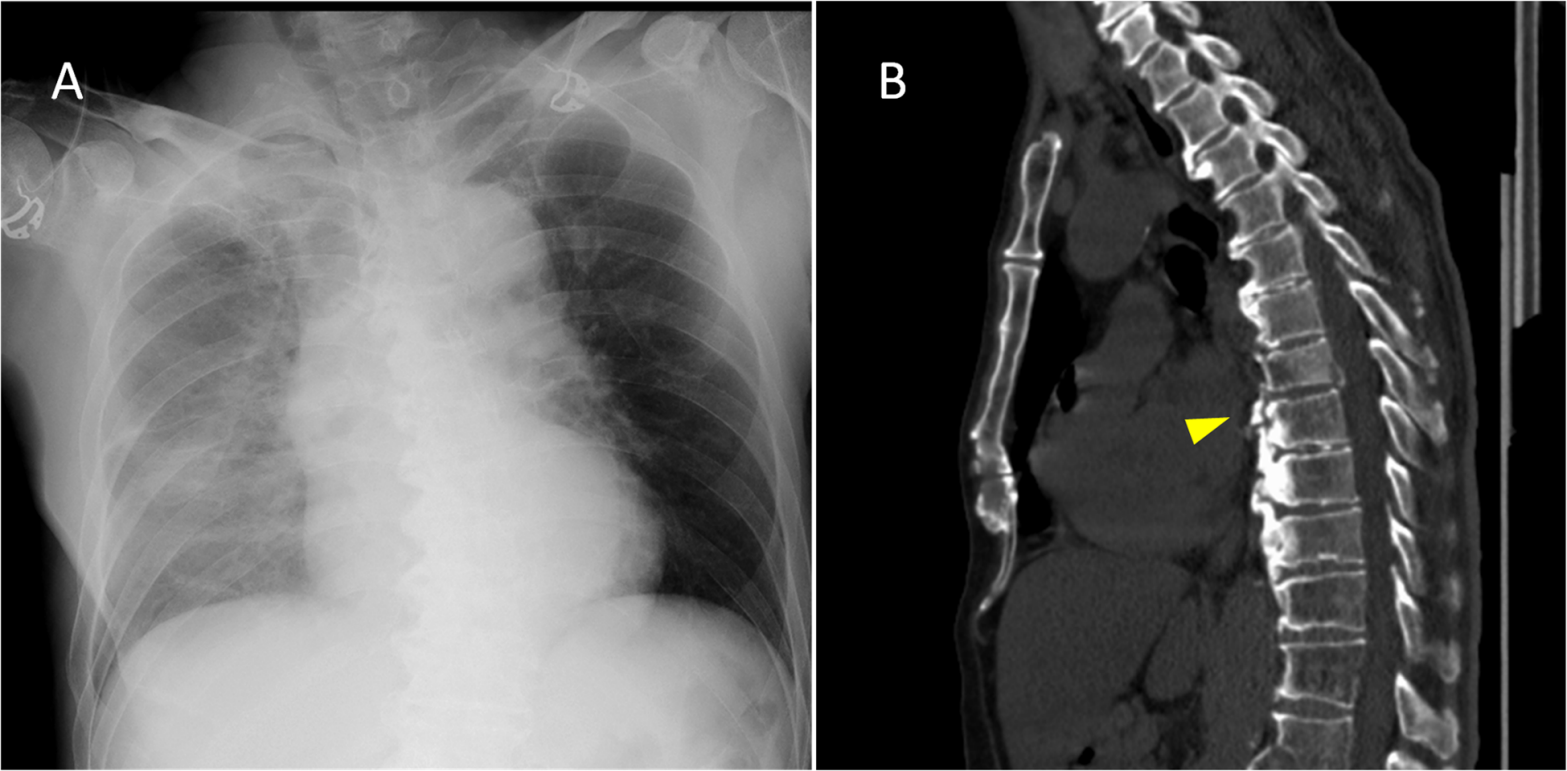
Images from Case 2: **a** Chest radiography shows a diffuse opacity of the right chest, and **b** computed tomography shows a T7-burst fracture (arrow head) and L1 fracture

A 64-year-old man was hospitalized after a fall from height. Initially, he was alert and hemodynamically stable but suddenly deteriorated after being transferred back from the CT room; CT scanning revealed TBI, right multiple rib fractures, right hemopneumothorax, and burst fractures of T7 and L1 (Fig. 2). Ultrasonography showed a gradually increasing hemothorax on the right side, and a right tube thoracostomy was performed. Approximately 1300 mL of blood was drained within a minute with persistent hemodynamic instability; therefore, an ERT was conducted to identify the bleeding foci. Active bleeding from a T7-burst fracture was detected on direct vision, but no other source of bleeding was identified during a right anterolateral thoracotomy. DCS with bone wax, hemostatic agent (*TachoSil*®), and gauze packing was effective to ensure hemostasis, but bleeding recurred during transfer to the ICU. A re-thoracotomy was conducted, and we identified another bleeding site in the breach of a T7-burst fracture, which was similarly treated. Re-exploration surgery was conducted 24 h after resuscitation, and the chest was closed after confirming hemostasis, and an ISS of 29 was recorded. The patient required treatment of catheter-related bloodstream infection. Spinal fixation with percutaneous pedicle screw was undertaken on the 10th day of hospitalization. The patient developed pneumonia and remained in the ICU for 16 days, after which he was shifted to the ward and subsequently transferred to another hospital on Day 40 of hospitalization.

#### Systematic review

**Fig. 3:**
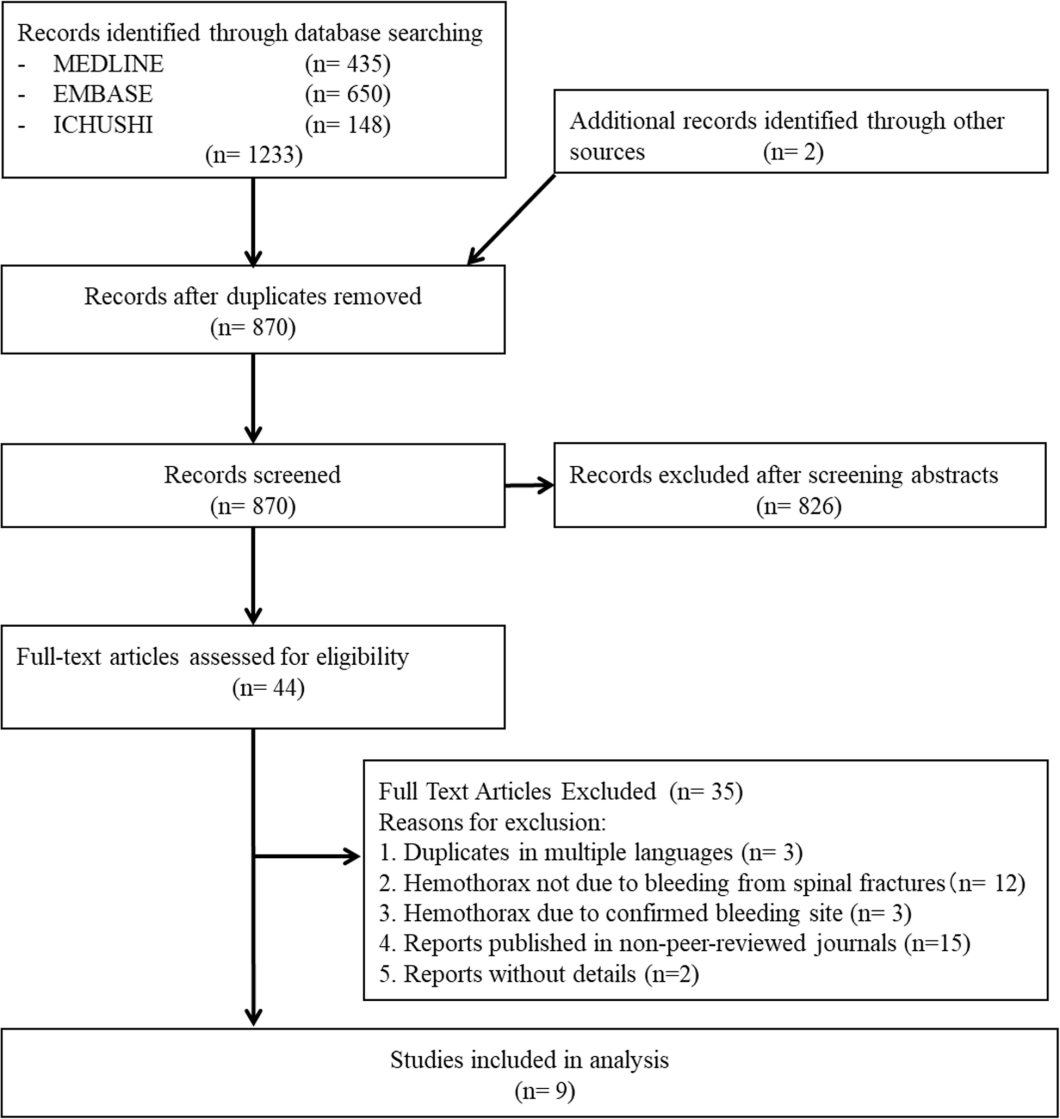
Study selection

Our search generated 1235 titles and abstracts. After screening and application of our inclusion criteria, only 9 articles (six in English and three in Japanese) were included (Fig. 3) [8–16]. Details of 12 cases, including the above-described patients, are shown in Table 1. The median age was 74 (range 28–93) years, and seven (58.3%) patients were men. The commonest mechanism of injury was a fall (*n* = 5; 41.7%), followed by traffic accidents (*n* = 4; 33.3%). The inferior part of the thoracic spine was more likely to be injured (T11 fracture in five cases; 41.7%). All patients had high spinal instability, although the incidence of neurological deficit was unclear because of missing data. Some cases had a history of osteoporosis (*n* = 3) and diffuse idiopathic skeletal hyperostosis (*n* = 3). Most patients went into hemorrhagic shock earlier or later in their clinical course. Three cases developed intra-transfer hemorrhages in the hospital. Some other cases reported the hemodynamic deterioration during the transfer to the hospital. In all cases, right-sided hemothorax was observed and a right thoracostomy revealed massive, active bleeding (median blood loss, 1400 [range, 1000–3000] mL). Moreover, bilateral hemothorax was observed in three cases, although critical hemothorax occurred on the right side in all cases. In 10 cases, after excluding two cases wherein best supportive care was chosen, the majority of hemostatic procedures included direct compression (*n* = 8); spinal fixation for hemostasis was done in only one case. A combination of gauze packing, bone wax, and hemostatic agents was used in most cases. The mortality rate in this patient population was 33.3% (4/12 cases). In five cases where detailed information was available, pneumonia was the major complication (*n* = 4), followed by catheter-related bacteremia (*n* = 1). In four cases, the length of hospital stay ranged from 33 to 130 days. Spinal fixation was undertaken in six cases, including our case [8–10, 12, 16]. With the exception of one case wherein spinal fixation was done on the day of massive rebleeding as a definitive surgery [8], spinal fixation was undertaken as a planned definitive surgery between 1 and 10 days following hospitalization [9, 10, 12, 16].

**Table 1:** Clinical characteristics of 12 cases (10 reported cases and our 2 cases)

Author/ Year	Age	Sex	Injury mechanism	Thoracic spinal fracture as the bleeding source (site/ Fx type)	Other sites of injury	Initial hemodynamic status	Side of HTX	Drained volume of Right HTX (mL)	Intervention	Hemostatic technique	Deterioration during the transfer	Spinal fixation	Prognosis
Dalvie/ 2000 [8]	28	M	Traffic accident	T4 / dislocation	NR	NR	Bilateral	NR	Right thoracotomy	spinal fixation	Yes	Performed	Survived
van Raaij/ 2000 [13]	55	F	Fall	T11 / Chance	Compression Fx (T10 & 12), chest, pelvis, limb	unstable	Right	1500	Right thoracotomy	bone wax, synthetic patch	NR	NR	Survived
Lu/ 2010 [10]	72	F	Traffic accident	T11 / burst + T12	NR	unstable	Bilateral	1300	Right thoracotomy	bone wax, gauze packing	NR	Performed	Survived
Masteller/ 2012 [11]	93	M	Fall	T10–11 / burst	chest, L1–2 Fx	NR	Right	1000	Only thoracentesis.	–	NR	Not performed	Dead
Masteller/ 2012 [11]	71	M	Transfer in OR	T11 / burst	none	unstable (CPA later)	Right	3000	Only thoracentesis.	–	NR	Not performed	Dead
Okamoto/ 2018 [12]	81	M	Fall	T7 / Chance	NR	stable (unstable later)	Right	1330	Right thoracotomy	bone wax, coagulant sheet	NR	Performed	Survived
Hirota/ 2019 [9]	74	F	Fall	T11 / Chance	none	unstable	Right	1200	Right thoracotomy	coagulant sheet	NR	Performed	Survived
Kaneko/ 2000 [14]	86	F	Unclear	T6 / dislocation	NR	unstable	Right	2000	Right thoracotomy	argon beam, iliopsoas muscle flap	NR	Not performed	Dead
Matsushita/ 2016 [16]	67	M	Hit by a lumber	T3 / dislocation	chest, T12 Fx (dislocation), limb	unstable	Bilateral	2090	Right thoracotomy	coagulant sheet	NR	Performed	Survived
Haruta/ 2016 [15]	78	F	Traffic accident	T8 / reverse Chance	TBI, chest, liver, pelvis	unstable (CPA later)	Right	1400	Left thoracotomy followed by clamshell thoracotomy	gauze packing, coagulant sheet	NR	Not performed	Dead
Our case	81	M	Traffic accident	T8 / burst	TBI, C5 Fx, chest, pelvis, limbs	unstable	Right	1500	Right thoracotomy	gauze packing	Yes	Not performed	Survived
Our case	64	M	Fall	T7 / burst	TBI, chest, L1 Fx	stable (unstable later)	Right	1300	Right thoracotomy	gauze packing, bone wax, coagulant sheet	Yes	Performed	Survived

Abbreviations: *C* cervical spine, *CPA* cardiopulmonary arrest, *F* female, *Fx* fracture, *HTX* hemothorax, *L* lumber spine, *M* male, *NR* not reported, *OR* operation room, *T* thoracic spine, *TBI* traumatic brain injury

## Discussion

A review of medical records suggested that the incidence of hemothorax due to bleeding from spinal fracture is 0.3% in all chest trauma and 0.9% in all hemothorax cases. These two cases indicated that spinal fractures can cause lethal hemothorax. Both cases had right-sided chest opacity on chest radiographs; however, it is difficult to understand what transpired between the radiography and physical examination. An ERT was undertaken for hemodynamic instability in both patients and resulted in a diagnosis of hemothorax secondary to spinal fractures. As the bleeding site was a breach of the thoracic vertebrae, the hemorrhage was uncontrollable by ligation or suturing. Sealing with bone wax was temporarily effective; however, DCS and direct compression were needed for hemostasis.

Our systematic review provides some insights into the characteristics of the clinical entity posed by thoracic spinal fracture-induced hemothorax. First, all cases had right-sided massive hemothorax, which necessitated right thoracotomy for hemostasis. Some anatomical differences may explain this phenomenon. The aorta exists on the left anterolateral side of the thoracic spine and covers part of the vertebral body, but no organ covers the right side of thoracic spine, rendering the spine susceptible to injury, and hemorrhage from the thoracic spine can easily enter the pleural cavity. Second, all cases had unstable spinal fractures. The bleeding occurred from a breach in the fractured spinal column; thus, high instability of the injured spinal bone indicates a larger cleavage of the spinal bones and, therefore, the amount of bleeding could be greater in such patients. Third, patient transfer can confer a risk of injury exacerbation and resultant hemorrhage. In three cases, bleeding recurred during the transfer. Therefore, shear stresses on the vertebral cleavage could have caused bleeding. Transfer to the Interventional Radiology suite or the operating theater can induce sudden deterioration. Lastly, the commonest complication was pneumonia. This clinical entity often involves costal injuries (3/4 cases), necessitating caution with regard to pneumonia as an important complication, consistent with thoracic injuries and thoracic spinal injuries. Empyema and osteomyelitis, although not observed in our review, can theoretically pose complications, especially in cases that require ERT. Future case reports or series need to evaluate details of hemothorax-induced complications for prediction of prognosis with the patient or their family.

**Fig. 4:**
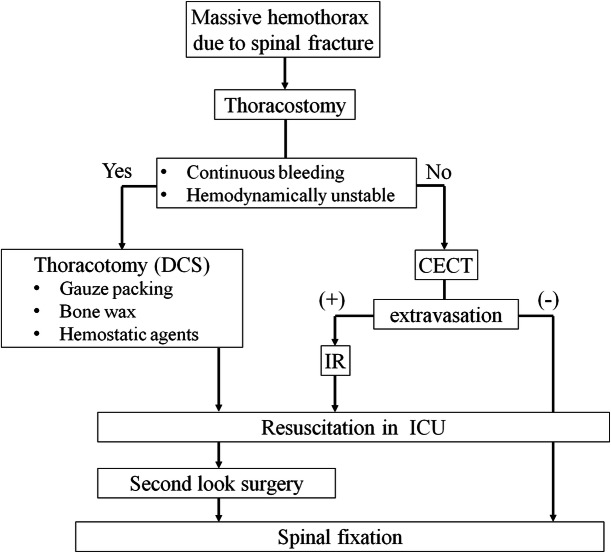
Our proposed treatment algorithm for massive hemothorax due to bleeding from a fracture of the thoracic spine. Abbreviations; CECT, contrast-enhanced computed tomography; DCS, damage-control surgery; ICU, intensive care unit; IR, interventional radiology

Initial management should follow the “Primary Survey” recommended in the Advanced Trauma Life Support (ATLS) approach, which prioritizes physiological status rather than anatomical problems. In the ATLS approach, thoracostomy is recommended as an initial treatment for massive hemothorax. However, for moribund patients with an obvious massive hemothorax, emergency thoracotomy is the only treatment option, given that further investigations, including CT angiography, are not possible because it requires patient transfer and consumes additional time. As suggested by our systematic review, spinal bleeding can be exacerbated because of the instability of fractured spines; therefore, emergency spinal fixation may be an ideal treatment of choice. However, restoration of physiological homeostasis is the primary focus of the treatment strategy, and the emergency spinal fixation for the patients with physiologically unstable states, such as coagulopathy, acidosis, and hypothermia, should be avoided. These patients cannot withstand transfer to the operating theater, prone positioning, and prolonged surgery. In such patients, elective spinal fixation should be planned after physiological recovery in the ICU. Right-sided thoracotomy may be needed because lethal hemothorax due to bleeding from the spinal fracture usually occurs into the right thorax. The bleeding site is often a breach of parietal pleura and fractured vertebral body, where suturing is difficult; therefore, direct compression appears to be the only approach for hemostasis. Gauze packing, bone wax, and some hemostatic agents may be useful; however, it is noteworthy that definitive hemostasis is impossible in most cases. If the patient’s condition suddenly deteriorates after the first thoracotomy, reoperation should be considered for the assessment of re-bleeding. In patients who are hemodynamically stable, contrast-enhanced CT is useful to plan treatment. If extravasation is detected, interventional radiology such as arterial embolization can be an option [4, 17]. A treatment algorithm for patients with hemothorax is presented in Fig. 4. The goal of initial management is to stop bleeding, restore blood volume by transfusion, and watchfully wait for the crisis to pass.

All patients had highly unstable spinal fractures, but the timing of complete spinal fixation is unclear. The concept of early total care (ETC), which indicates early definitive stabilization of major skeletal injuries, was proposed for femoral fractures in the 1970s. Thereafter, the idea of damage-control orthopedics (DCO) for multiple severe trauma arose [18] and involves a stepwise approach based on the concept of DCS. It comprises initial hemostasis and a planned secondary definitive procedure. Some systematic reviews suggested advantages of ETC, such as shorter length of ICU stay, shorter length of hospital stay, fewer days of mechanical ventilation, and lower pulmonary complications and sepsis, even in spinal fractures [19, 20]. However, ETC is unadvisable in cases with physiological derangement. A prospective cohort study showed higher ETC-associated mortality in a cohort of polytrauma patients with severe thoracic trauma (ISS > 15), particularly in patients who subsequently needed thoracostomy and had low initial hemoglobin levels (< 10 mg/dL). Therefore, ETC should be chosen carefully [21]. From our experience, ETC may be impossible because the patient may be dying of exsanguination and may need a massive transfusion; therefore, an intervening resuscitation period is essential. The literature review revealed six cases that underwent spinal fixation. Dalvie et al. reported a single case that underwent emergency spinal fixation, but the report lacked information on the initial hemodynamic status. The other five cases underwent elective spinal fixations 1–10 days post thoracotomy. Thus, most cases were apparently stable enough to undergo elective spinal fixation or resuscitation until physiological improvement was prioritized. Recently, ETC has emerged as an important concept in the management of orthopedic trauma patients, although it should be considered only when the patient’s physiological status allows. The concept of safe definitive surgery (SDS), which was proposed by Pape, seems to be useful for decision-making [22] and is the pivotal concept of ETC and DCO, with repeated reevaluation of the patient’s physiological status. In contrast, the mortality of those who did not undergo spinal fixation was 80% (4/5 cases), including: a case by Haruta et al. where the patient was nonresponsive to resuscitation and died in the trauma bay [15]; two cases by Masteller et al. who received best supportive care [11]; and one by Kaneko et al. who suffered a cardiopulmonary arrest 2 days post thoracotomy and died of pneumonia 33 days after the thoracotomy [14]. These outcomes suggest that the patients had an unstable physiological condition and could not receive spinal fixation during the clinical course. Given the diversity of trauma patients, individualized treatment policies are needed.

This study has some limitations. First, although we reviewed all trauma cases in our institution during the study period, we may have missed some relevant cases. The diagnosis could only be confirmed under direct visualization by thoracotomy; patients who survived without thoracotomy or died without autopsy might have been missed in our database review. Masteller et al. reported two similar cases that were diagnosed by autopsy [11]. Moreover, we may have missed patients with small, self-limited hemothorax-induced spinal fractures owing to an inability to identify the bleeding source. Second, some relevant data are missing despite our efforts to contact the original authors. Similar case reports need to be described in the future, particularly with regard to complications and prognosis, to provide relevant information for clinical decision making in the management of patients with hemothorax-induced spinal fractures. Third, although we conducted a comprehensive literature search, similar cases may not have been unpublished. In patients who died of this condition, the clinicians may have chosen not to publish case reports, which could potentially confer publication bias. As this condition is extremely rare, a collection of similar cases is needed to ensure better recognition and understanding of this disease entity.

## Conclusions

Massive hemothorax due to thoracic spinal fracture is rare but can be an unexpected and fatal hemorrhagic source that tends to occur on the right side. Moreover, the inferior part of the thoracic spine is likely to be injured. Patient transfer may confer additional risk of hemorrhage. Most patients suffer hemodynamic instability, and thoracotomy with direct compression may be needed as DCS.

## Supplementary information

**Additional file 1.**

## Data Availability

All data generated or analyzed during this study are included in this published article.
