# Supplementary material for: Massive hemothorax due to bleeding from thoracic spinal fractures: a case series and systematic review
Source: Scand J Trauma Resusc Emerg Med. 2020 Sep 11;28:92. doi: 10.1186/s13049-020-00783-0 (PMC7488342; doi:10.1186/s13049-020-00783-0)
Supplement: Supplementary file 1 — Additional file 1. [file 13049_2020_783_MOESM1_ESM.docx]

**Supplementary Table 1**. Search strategy for Embase

#1. 'lumbar vertebra' OR 'thoracolumbar spine' OR 'thoracic spine' OR 'lumbar spine'

#2. thoraco*:ti,ab,tn OR thoracic:ti,ab,tn OR lumbar:ti,ab,tn

#3. #1 OR #2

#4. 'spine fracture' OR 'vertebra fracture' OR 'fracture' OR fractur*:ti,ab,tn

#5. 'hemothorax'/exp OR 'haemothorax'/exp

#6. 'haemorrhagic pleural effusion' OR 'hemorrhagic pleural effusion'

#7. 'hemopneumothorax'/exp OR 'haemopneumothorax'/exp

#8. 'haematopneumothorax'/exp OR 'hematopneumothorax'/exp

#9. #5 OR #6 OR #7 OR #8

#10. #3 AND #4 #9
